# Subclinical Cardiovascular Disease Markers in Relation to Serum and Dietary Magnesium in Individuals from the General Population: The KORA-MRI Study

**DOI:** 10.3390/nu14234954

**Published:** 2022-11-22

**Authors:** Nuha Shugaa Addin, Christopher L. Schlett, Fabian Bamberg, Barbara Thorand, Jakob Linseisen, Jochen Seissler, Annette Peters, Susanne Rospleszcz

**Affiliations:** 1 Institute for Medical Information Processing, Biometry, and Epidemiology (IBE), Ludwig-Maximilians-Universität (LMU), 85764 München, Germany; 2 Institute of Epidemiology, Helmholtz Zentrum München, German Research Center for Environmental Health, 85764 Neuherberg, Germany; 3 Department of Diagnostic and Interventional Radiology, University Medical Center Freiburg, Faculty of Medicine, University of Freiburg, 79106 Freiburg, Germany; 4 German Center for Diabetes Research (DZD), 85764 Neuherberg, Germany; 5 Epidemiology, University Hospital of Augsburg, University of Augsburg, 86159 Augsburg, Germany; 6 Diabetes Zentrum, Medizinische Klinik und Poliklinik IV, Klinikum der Ludwig-Maximilians-Universität München, 80336 München, Germany; 7 German Centre for Cardiovascular Research (DZHK e.V.), Partner Site Munich Heart Alliance, 80802 München, Germany

**Keywords:** magnesium, preclinical atherosclerosis, early cardiac impairment, cardiac MRI

## Abstract

Several studies have implied a role of magnesium in the development of cardiovascular disease (CVD). Thus, magnesium might serve as a potential risk marker for early CVD. Therefore, we investigated the association of serum magnesium and dietary magnesium intake with markers of subclinical CVD in a population-based study. We used cross-sectional data from the sub-study of the Cooperative Health Research in the Region of Augsburg (KORA-FF4). Markers of subclinical CVD, namely, left and right ventricular structure and function and carotid plaque and carotid wall thickness, were derived by magnetic resonance imaging (MRI). Multivariable-adjusted regression models were applied to assess the relationship between serum and dietary magnesium and MRI-derived subclinical CVD markers. Among 396 included participants (mean age: 56.3 ± 9.2 years; 57.8% male), 181 (45.7%) had low serum magnesium levels (<2.07 mg/dL). Among 311 subjects with complete dietary data (mean age: 56.3 ± 9.1 years; 56.3% male), 154 (49.5%) had low dietary magnesium intake (≤155.2 mg/1000 kcal/day). Serum and dietary magnesium were not correlated (*p*-value = 0.5). Serum magnesium was significantly associated with presence of carotid plaque (OR 1.62, *p*-value 0.033). Dietary magnesium was associated with higher left ventricular end-systolic and end-diastolic volume (0.04 mL/m^2^, 0.06 mL/m^2^; *p*-value 0.011, 0.013, respectively), and also with a decrease in left ventricular remodeling index and mean diastolic wall thickness (−0.001 g/mL/m^2^, −0.002 mm/m^2^; *p*-value 0.004, 0.029, respectively). In summary, there was no consistent association of serum and dietary magnesium with imaging markers of subclinical CVD.

## 1. Introduction

Cardiovascular disease (CVD) is a major contributor to reduced quality of life and a leading cause of mortality worldwide [1,2]. The Global Burden of Disease (GBD) Study 2019 showed that the prevalence, mortality, and disability-adjusted life years (DALYs) of CVD have risen significantly since 1990 [2]. Heart failure prevalence is projected to rise by 40% between 2015 and 2035 [3]. The global prevalence of carotid plaque, an indicator of cardiovascular risk, was estimated to be 21.1% in people aged 30–79 years in 2020 [4]. However, the prevalence of CVD and plaque burden cannot be solely explained by the traditional cardiovascular risk factors such as hypertension and diabetes [5,6]. Thus, there is an urgent need to identify additional markers of early CVD in order to implement prevention and treatment strategies to limit the growing burden of CVD.

One potential candidate is magnesium, which has been reported to be implicated in CVD development [7,8]. As the fourth most abundant mineral and the second most abundant intracellular cation, magnesium modulates neuronal excitation, intracellular conduction, and myocardial contraction [9]. Furthermore, magnesium plays a key role in regulating mitochondrial function and energy production [10]. Liu et al. found that magnesium deficiency can induce diastolic cardiomyopathy in mice with a low magnesium diet mainly through ATP depletion, mitochondrial dysfunction, and overproduction of reactive oxygen species [11]. Magnesium deficiency has also been shown to accelerate atherosclerosis by promoting platelet activity and endothelial dysfunction and increasing the production of pro-inflammatory cytokines and neuropeptides [12].

Dietary magnesium is deemed to be one of the shortfall nutrients due to the modern Western diet characterized by a wide use of processed foods, demineralized water, and agricultural practices that use soils deficient in magnesium [13]. A meta-analysis of prospective cohort studies found that increasing dietary intake of magnesium was associated with a 22% reduction in the risk of heart failure [14]. Low magnesium intake was associated with higher heart failure incidence and hospitalization [15,16]. However, to assess if magnesium is a potential target for CVD prevention, it is necessary to study its relationship with early subclinical CVD, which could be assessed with cardiovascular imaging.

Using echocardiography, serum magnesium was inversely associated with left ventricular mass even after adjustment for cardiovascular risk factors [17]. Previous population-based studies have demonstrated an inverse relationship between serum magnesium and carotid artery intima-media thickness [18,19,20]. On the other hand, a systematic review and meta-analysis of randomized clinical trials showed that magnesium supplementation may improve endothelial function without affecting carotid intima-media thickness [21]. However, intima-media thickness is not a good measure of atherosclerotic plaque [22]. Cardiac magnetic resonance imaging (MRI) is considered a safe, non-invasive, highly accurate, and reproducible method that enables a detailed characterization of cardiac morphology and function and atherosclerotic plaque [23]. We now aim to make use of the detailed cardiac MRI data as measures of subclinical CVD and analyze the association between serum and dietary magnesium with early cardiac impairment and preclinical atherosclerosis.

## 2. Materials and Methods

### 2.1. Study Design and Population

We used cross-sectional data from a subsample (KORA-MRI, n = 400) of a population-based cohort in southern Germany (KORA-FF4, n = 2279). KORA-FF4 is the second follow-up of the original baseline survey KORA-S4 (N = 4261, enrolled between 1999 and 2001). Details on the study design, sampling method, and data collection of the KORA surveys have been described elsewhere [24]. In the KORA-MRI sub-study, a total of 400 participants aged 39 to 73 years without known cardiovascular disease underwent whole-body MRI. The main aim of the study was to assess subclinical disease in individuals with prediabetes and diabetes. Study setup, imaging protocol, and inclusion and exclusion criteria were described in detail previously [25].

The KORA-FF4 study was approved by the Ethics Committee of the Bavarian Medical Association (Bayerische Landesärztekammer). All investigations were performed in accordance with the Declaration of Helsinki, including written informed consent of all participants. The MRI examination protocol was further approved by the ethics committee of the Ludwig-Maximillian-University Hospital, Munich.

### 2.2. Assessment of Serum and Dietary Magnesium

Plasma magnesium was measured in mmol/L with the Siemens Dimension Vista (Siemens Health Care Diagnostics Inc, Newark, DE) using a modification of the methyl thymol blue (MTB) procedure that forms a blue complex with magnesium [26]. The amount of magnesium-MTB complex formed is proportional to the magnesium concentration and was measured using a biochromatic (600 and 510 nm) endpoint technique. Based on a review of the literature that adopted an evidenced-based reference interval for serum total magnesium concentration, we used a cutoff point of <0.85 mmol/L (2.07 mg/dL) to indicate hypomagnesemia [27].

Habitual dietary intake, including magnesium, was obtained based on a combination of up to three 24 h food lists (24H-FL) and a food frequency questionnaire (FFQ), as described previously [28]. While the 24H-FL, consisting of 246 food items, was used to calculate the consumption of foods over the previous day, the FFQ, consisting of 148 food items, was used to assess the dietary habits in the past 12 months. For each participant, the assessment of the usual dietary intake of each food item on any given day was derived from the multiplication of the calculated consumption probability and consumption amount. Consumption probability was determined by logistic mixed models adjusted for covariates and FFQ information and the consumption amount was estimated based on data from the Bavarian Food Consumption Survey II (BVS II). According to the EPIC-Soft classification scheme, food items were combined into 16 food groups and 21 subgroups for both approaches. Dietary magnesium was measured in mg/day. In order to take the total energy consumption into account, we dichotomized dietary magnesium intake based on the median split of the dietary density for dietary magnesium. A magnesium intake ≤ 155.2 mg/1000 kcal/day was defined as low.

### 2.3. Assessment of Subclinical Cardiovascular Disease Markers by MRI

Whole-body MRI examinations were performed using a 3 Tesla Magnetom Skyra (Siemens AG, Healthcare Sector, Erlangen, Germany) supplied with an 18-channel body coiling system. All participants underwent imaging, within 3 months after their clinical examination at the study center, consisting of sequences covering the entire body. For analysis of the heart, 4-chamber view steady-state free precession (SSFP) and short-axis stack SSFP were used. An axial black-blood T1 weighted fat-saturated (T1w fs ax) was used to assess carotid plaque. All image analyses were performed by independent readers blinded to the clinical covariates of the participants [25]. Missing values in MRI parameters were due to technical malfunction, low image quality, or imaging artifacts.

#### 2.3.1. Left Ventricular Structure and Function

Cine-SSFP sequences were evaluated semi-automatically with cvi42 (Circle Cardiovascular Imaging, Calgary, Canada) software. The derived left ventricular (LV) markers [29] included end-diastolic volume (the phase with the biggest left ventricular volume), end-systolic volume (the phase with the smallest left ventricular volume), stroke volume (end-diastolic volume minus end-systolic volume), ejection fraction ((stroke volume/end-diastolic volume) * 100), left ventricular mass assessed during end diastole, cardiac output (left ventricular stroke volume * heart rate), and left ventricular wall thickness. Presence of late gadolinium enhancement (LGE) was assessed visually on fast low-angle shot inversion recovery sequences. Furthermore, filling and ejection rates were quantified using dedicated in-house software estimating peak gradients during early (passive left ventricular filling) and late (left ventricular filling due to atrial contraction) filling [30].

#### 2.3.2. Right Ventricular Structure and Function

Right ventricular (RV) function was assessed by manual segmentation of the right ventricular endocardial border on axial cine-SSFP sequences using dedicated software (cvi42, Circle Cardiovascular Imaging, Calgary, Canada). Markers included end-systolic volume, end-diastolic volume, stroke volume, cardiac output, and ejection fraction [31].

#### 2.3.3. Carotid Plaque

The presence and composition of carotid plaque was determined on black-blood T1-weighted sequences on 14 slice positions and semiautomatic software (CASCADE; University of Washington Seattle, WA) was used to obtain vessel wall thickness and lumen dimension [32]. According to plaque composition [33], measures were classified as type I, type III, type IV/V, and type VI/VII plaques, and any plaque > type I was defined as having plaque.

### 2.4. Assessment of Covariates

All participants underwent standardized interviews, comprehensive medical examinations, and a fasting blood draw at the study center. Interviews included information on demographic variables (e.g., age, sex), medication intake (e.g., antihypertensive and antidiabetic medication), and health behavior (e.g., smoking and physical activity). Smoking status was classified as never smoker, ex-smoker, and current smoker. Height, weight, BMI, and waist circumference were assessed by trained staff according to standard protocols using standardized instruments. Systolic and diastolic blood pressure was measured three times on the right arm of seated participants after at least a five-minute resting period. The mean of the second and third BP measurements was used for the analyses. Hypertension was defined as blood pressure greater or equal to 140/90 mmHg or the use of antihypertensive medication under the awareness of having hypertension.

Diabetes status (normoglycemia, prediabetes, or diabetes) was determined by an oral glucose tolerance test (OGTT) according to WHO criteria [34] or as previous diabetes diagnosed by a physician. Laboratory parameters, such as glucose, HbA1c as well as total cholesterol, triglycerides, and low- and high-density lipoprotein cholesterol were assessed by standardized methods as described elsewhere [35].

### 2.5. Statistical Analyses

Participants’ demographics, cardiovascular risk factors, and MRI outcomes are presented as means and standard deviations (SD) for continuous variables and counts and percentages for categorical variables. Description was also stratified by serum and dietary magnesium with cut-off points of 0.85 mmol/L (2.07 mg/dl) and 155.2 mg/1000 kcal/day, as outlined above. Differences in continuous and categorical variables between the groups were examined by a *t*-test and χ^2^-test, respectively. We calculated the body surface area (BSA) according to the Du Bois formula (BSA [m^2^] = weight [kg]^0.425^ × height (cm)^0.725^ × 0.007184) [36] and indexed all measures of cardiac morphology and function by BSA. Correlations between serum and dietary magnesium and correlations with MRI outcomes were determined by Spearman’s rho correlation coefficient and corresponding *p*-value.

To assess the association between continuous exposures of serum and dietary magnesium with continuous MRI outcomes, a linear regression model adjusted for age, sex, BMI, systolic blood pressure, and diabetes status was calculated providing β-coefficients with 95% confidence intervals (CIs). Serum magnesium was standardized by subtracting its mean and dividing by its SD. For dietary magnesium intake, models were additionally adjusted for daily caloric intake. Binary MRI outcomes (presence of LGE and presence of plaque) were analyzed by logistic regression adjusted for the same variables. Categorical MRI outcome (plaque-type) was analyzed by ordered logistic regression with a cumulative logit link under the proportional odds assumption. As an additional analysis for serum magnesium, logistic models were additionally adjusted for serum total cholesterol and smoking status. Estimates for all logistic models are reported as odds ratios (ORs) with 95% CIs. To assess if the association of serum magnesium with presence of plaque was mediated by serum total cholesterol, causal mediation analysis was applied. Due to the varying number of missing values in the MRI parameters, we performed complete-case analyses for each exposure and each MRI outcome. *p* values < 0.05 were considered to indicate statistical significance. All analyses were conducted with R (Version 4.1.2).

## 3. Results

### 3.1. Study Population

**Figure 1:**
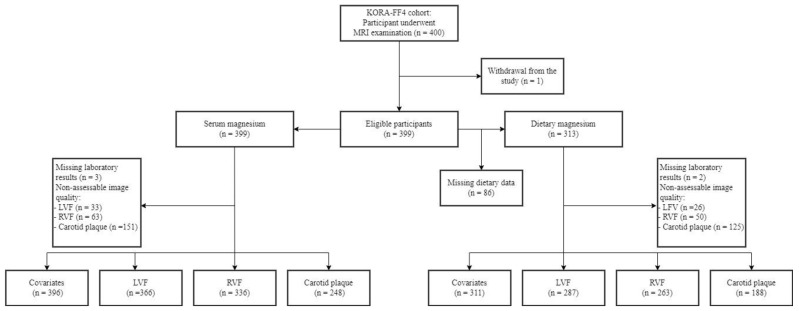
Participant flow diagram. LVF, left ventricular function; RVF, right ventricular function.

Samples sizes for the analyses of serum and dietary magnesium with the respective MRI outcomes of LV structure and function, RV structure and function, and carotid plaque are presented in Figure 1. Among the 400 participants who underwent whole-body MRI, 396 and 311 subjects were included in the analysis serum and dietary magnesium, respectively. Data on the relationship between serum magnesium and subclinical cardiovascular disease markers were available in 363, 333, and 245 participants for LV, RV, and carotid plaque, respectively. Similarly, the association between dietary magnesium and subclinical cardiovascular disease markers was available in 287, 263, and 188 individuals for LV, RV, and carotid plaque, respectively. Differences in the clinical characteristics of the study population between missing and complete case analysis are presented in Table S1.

Table 1a provides an overview of the clinical characteristics of the study population stratified by serum magnesium levels. Out of 396 included participants (mean age: 56.3 ± 9.2 years; 57.8% male), 181 (45.7%) had low serum magnesium levels (mean age: 57.04 ± 9.65 years; 59.7% male). Individuals with hypomagnesemia had higher systolic blood pressure, more often had diabetes, had higher blood glucose levels, with a more frequent use of diabetic medications.

Table 1b shows the clinical characteristics of the study participants stratified by dietary magnesium intake. Among 311 subjects with complete dietary data (mean age: 56.3 ± 9.1 years; 56.3% male), 154 (49.5%) had low dietary magnesium intake (mean age: 55.9 ± 9.2 years; 76.6% male). Men were more likely to have significantly lower dietary magnesium intake in comparison to women (*p*-value <0.001). Furthermore, individuals with low dietary magnesium intake were more likely to have higher body weight, waist circumference, glucose levels, systolic and diastolic blood pressure, triglycerides, energy intake, and lower HDL cholesterol and dietary calcium intake.

Concordance between dichotomized serum magnesium and dichotomized dietary magnesium is shown in Table S2. Among 311 participants, 75 (24.1%) had both low serum and dietary magnesium and 91 (29.3%) had both high serum and dietary magnesium.

Overall, mean MRI markers of subclinical cardiovascular disease within the study sample were within normal limits (Table 2a,b).

### 3.2. Correlation between Serum and Dietary Magnesium with Clinical Characteristics and MRI-Derived Markers

**Figure 2:**
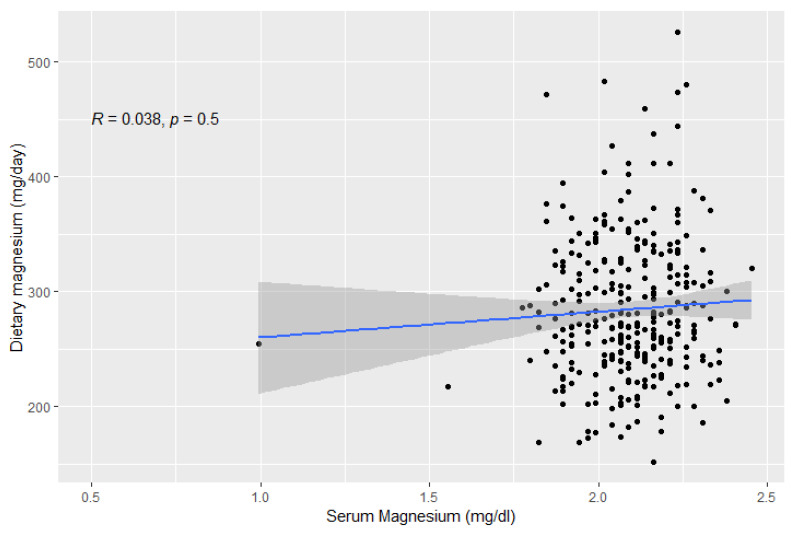
Correlation between serum and dietary magnesium, as assessed by Spearman correlation.

In our sample, there was no correlation between serum and dietary magnesium (Spearman’s rho = 0.04, *p* = 0.5, Figure 2). Serum magnesium was negatively correlated with systolic blood pressure and fasting blood glucose (*p* = 0.003, Table S3), and positively correlated with total cholesterol and LDL-cholesterol (*p* < 0.05, Table S3). Regarding MRI outcomes, serum magnesium was negatively correlated with right carotid artery wall thickness (*p* = 0.037), but not with any other MRI parameter of cardiac structure and function. On the other hand, dietary magnesium intake was positively correlated with systolic blood pressure and body weight (*p* < 0.05, Table S3). Expectedly, dietary magnesium was highly correlated with dietary potassium and dietary phosphate (Table S3). Regarding MRI outcomes, dietary magnesium was positively correlated with LV and RV end-systolic and end-diastolic volume and myocardial mass, and negatively correlated with ejection fraction, peak ejection rate, remodeling index, and mean diastolic wall thickness.

### 3.3. Association between Magnesium and MRI-Derived Subclinical Cardiovascular Disease Markers

#### 3.3.1. Serum Magnesium

In univariate analysis, markers of early cardiac impairment and preclinical atherosclerosis were similar between groups with and without hypomagnesemia (Table 2a). However, right ventricular end-systolic volume and right carotid artery wall thickness were significantly higher in the group with low serum magnesium levels (*p*-value: 0.031, 0.018, respectively). Multivariable analysis adjusted for age, sex, BMI, systolic blood pressure, and diabetes status revealed a significant negative association of continuous serum magnesium with right ventricular end-systolic volume (coefficient: −1.21mL/m^2^; 95% CI −2.39mL/m^2^ to −0.04mL/m^2^). Higher ORs for LGE, presence, and type of carotid plaque were also observed, which remained significant even after additionally adjusting for serum cholesterol and smoking status (OR = 3.06, 95% CI 1.27 to 8.32; OR = 1.62, 95% CI 1.07 to 2.56; OR = 1.58, 95% CI 1.19 to 2.11, respectively) (Table 3). We note, however, that prevalence of LGE was low and this result has to be interpreted with caution.

**Figure 3:**
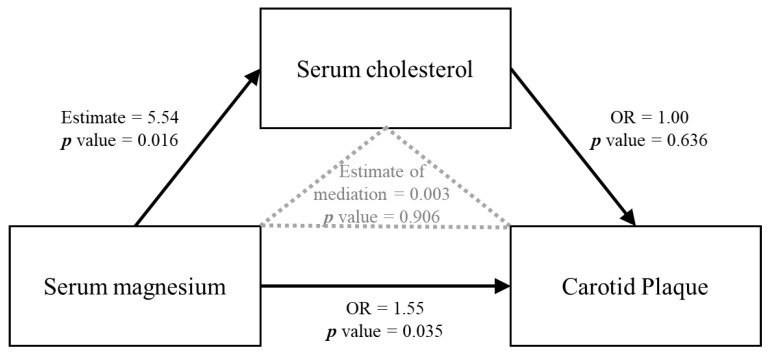
Direct acyclic graph (DAG) showing non-adjusted mediation analysis between serum magnesium, serum cholesterol, and carotid plaque.

In mediation analysis, the association of serum magnesium on carotid plaque was not mediated by serum cholesterol (coefficient: 0.003 mg/dl; 95% CI −0.06 mg/dl to 0.12 mg/dl) (Figure 3).

#### 3.3.2. Dietary Magnesium

Table 2b presents the univariate analysis between dietary magnesium intake and MRI markers of subclinical cardiovascular disease. Left ventricular myocardial mass was significantly higher in individuals with low dietary magnesium intake (73.57 g/m^2^ ± 12.56 g/m^2^ vs. 67.94 g/m^2^ ± 12.29 g/m^2^, *p*-value < 0.001). In addition, with a decrease in dietary magnesium intake, there was a decrease in right ventricular ejection fraction (52.37% ± 6.83% vs. 54.01% ± 6.88%, *p*-value < 0.001). In multivariable analysis, we observed a significant positive association between dietary magnesium intake and left ventricular end-systolic and end-diastolic volume after adjusting for age, sex, BMI, systolic blood pressure, and diabetes status (coefficients: 0.04 mL/m^2^, 0.06 mL/m^2^; CI 0.008 mL/m^2^ to 0.065 mL/m^2^, 0.014 mL/m^2^ to 0.114 mL/m^2^, respectively) (Table 3). Furthermore, there was an inverse association between dietary magnesium intake and left ventricular mean diastolic thickness and remodeling index (coefficients: −0.002 mm/m^2^, −0.001 g/mL/m^2^; CI −0.004 mm/m^2^ to −0.0002 mm/m^2^, −0.001 g/mL/m^2^ to −0.0002 g/mL/m^2^, respectively). Dietary magnesium was also associated with lower risk of more severe plaque (type of carotid plaque OR 0.99; 95% CI 0.98 to 0.99).

When stratifying the sample by both serum and dietary magnesium, no clear pattern in the distribution of MRI markers could be observed (Table S4).

## 4. Discussion

### 4.1. Main Findings

This comprehensive investigation of the association of serum and dietary magnesium with several imaging-derived markers of subclinical CVD burden in a population-based cohort revealed partly contradicting directions of results. While serum magnesium was associated with decreased right ventricular end-systolic volume, dietary magnesium intake was associated with increased left ventricular end-systolic and end-diastolic volume. Unlike dietary magnesium intake, serum magnesium was associated with a higher risk for carotid plaque. We also found an inverse association between dietary magnesium intake and left ventricular remodeling and mean left ventricular thickness. These associations were independent of sex, age, and common cardiovascular risk factors including hypertension and diabetes status.

### 4.2. Correlation between Serum and Dietary Magnesium

These inconsistent results will partly be due to the low correlation between dietary and serum magnesium (r = 0.038, *p*-value = 0.501). Only 75 subjects had consistently low serum and dietary magnesium and only 91 subjects had consistently high serum and dietary magnesium. The poor correlation between dietary magnesium and plasma levels of this mineral has been reported in several previous studies [37,38,39,40]. This could be explained by the fact that the magnesium concentration is regulated by a balance between intestinal absorption and kidney excretion. Any condition impairing intestinal absorption such as aging, inflammation, bowel disorders, or reduced kidney function could affect magnesium bioavailability and thus contribute to the lack of correlation between serum and dietary magnesium [41]. Furthermore, serum magnesium constitutes only 0.3% of the total body magnesium and thus may not necessarily reflect true body magnesium content. Indeed, magnesium concentration is under tight hemostatic regulation and can be maintained as normal, even if intakes are low, by reducing urinary excretion and mineral release from bone, muscles, and internal organs [42]. This is supported by the metabolic unit magnesium balance experiments on menopausal women, showing that serum magnesium levels did not significantly change with the magnesium depletion–repletion protocol. Furthermore, they found that consuming a magnesium-deficient diet for 72 to 92 days did not markedly decrease serum magnesium concentration [43]. This indicates that normal serum magnesium concentrations do not rule out magnesium deficiency.

### 4.3. Association of Magnesium with Overt and Early Cardiac Impairment

Magnesium has been implicated as a potential marker of CVD risk. In fact, evidence indicates that high serum and dietary magnesium are inversely associated with CVD [7,44]. There is a paucity of studies regarding the relationship of serum and dietary magnesium with early cardiac impairment markers. A population-based longitudinal study showed that low serum magnesium concentrations were associated with increased left ventricular mass [17]. The Jackson Heart Study found no association between quartiles of magnesium intake/Kg body weight and systolic function. However, dietary magnesium was inversely associated with Doppler peak mitral E wave velocity (a surrogate for diastolic function) and tricuspid regurgitation peak velocity (an estimate of pulmonary systolic pressures) [15]. Our results failed to demonstrate any association between serum magnesium and left ventricular parameters. On the other hand, we found an inverse association between serum magnesium and right ventricular end-systolic volume which could potentially imply a protective effect of serum magnesium to subclinical pulmonary vascular resistance. This is supported by an animal study which demonstrated that magnesium supplementation reduced pulmonary arterial pressure, right heart hypertrophy, and media wall thickness of pulmonary arteries [45].

Diastolic dysfunction is characterized by impaired myocardial relaxation and left ventricular filling and distensibility, which is associated with significant morbidity and mortality [46]. In our study, individuals with higher dietary intake of magnesium had larger end-diastolic volumes and less remodeling, indicating better diastolic function. These associations remained significant after multivariable adjustment. This is in good agreement with the Jackson Heart Study [15] as well as with an animal study showing an impaired relaxation with a decreased ratio between early and late diastolic velocity of the mitral valve in low-magnesium fed mice, which was reversed after magnesium repletion [11].

Notably, we observed increased rates of myocardial LGE—potential markers of minor myocardial infarction—in participants with high serum magnesium levels. It has been shown that in patients with acute coronary syndrome, there is a higher magnesium leakage from the infarcted myocardium leading to increased magnesium levels, with subsequent development of malignant ventricular arrhythmia and increased in-hospital deaths [47,48]. However, this cannot explain our findings since participants were not in an acute condition. We interpret our results with caution, since the absolute number of LGE was low.

It is crucial to note that despite the majority of observational studies favoring serum magnesium in the prevention of CVD, causal associations using Mendelian randomization remain inconsistent. One study found that a genetic predisposition to higher magnesium levels was inversely associated with coronary artery disease [49], whereas the other found no associations between serum magnesium and type 2 diabetes, coronary artery disease, heart failure, and atrial fibrillation [50]. Therefore, the effect of magnesium on the development of CVD remains equivocal.

### 4.4. Association of Magnesium with Lipids and Preclinical Atherosclerosis

The relationship between magnesium with preclinical carotid atherosclerosis is not novel, as this association has been described in different studies, using ultrasound for the assessment of carotid-intima media thickness as a proxy for atherosclerosis. While some studies found an inverse association between serum magnesium and common carotid intima-media thickness [8,18,19], others demonstrated an effect of magnesium supplementation on improving endothelial function but not carotid intima-media thickness [21]. A population-based study in Japan showed a significant association between low serum magnesium and mean intima-media thickness and risk of ≥2 carotid plaques [20]. Our results, however, suggested a positive association between serum magnesium and carotid plaque. On the other hand, higher dietary magnesium in the current study was modestly associated with decreased carotid plaque type. This is in good agreement with the study reporting that greater magnesium intake was associated with slightly lower odds of high common carotid artery intima-media thickness [51]. The accuracy of intima-media thickness as a marker of preclinical atherosclerosis is questioned since it can only measure the common carotid artery (laminal turbulent flow), rather than the internal carotid or carotid bulb. Furthermore, the main predictors of common carotid artery media hypertrophy are age and hypertension rather than atherosclerosis [22]. In contrast, carotid plaque has a better predictive ability of coronary artery disease. In fact, a meta-analysis of 11 population-based studies found that carotid plaque has a significantly higher diagnostic accuracy for the prediction of future myocardial infarction events in comparison to carotid intima-media thickness [52].

The relationship between magnesium status and dyslipidemia is ambiguous and studies present conflicting results. A study has suggested a possible magnesium–lipid interaction in atherosclerosis. They found that high LDL-cholesterol and triglyceride levels affect carotid intima-media thickness only when magnesium levels are low [53]. Nevertheless, we found that serum magnesium was positively associated with total cholesterol levels. This is consistent with previous research [8,18,19,20,54]. In causal mediation analysis, we found that the effect of magnesium on carotid plaque was not mediated by cholesterol. These results point to the theory suggesting a simple binding interaction between serum magnesium and lipoprotein particles rather than a complex physiological and pathological process [55].

### 4.5. Strengths and Limitations

The strengths of our study include the availability of both serum and dietary magnesium as markers of magnesium concentration in the body. The accurate measurement of various functional and structural parameters of subclinical CVD burden using an advanced MRI technique contrary to previous studies that used echocardiography and ultrasound was a strength of this study. Additionally, the well-designed population-based study that included extensive measurements and assessment of the dietary intake and different confounding variables was also a strength. Nevertheless, we are aware that our study has limitations that may have influenced the reported results. We had a relatively small sample size of participants which became more prominent in complete-case analyses. This will have reduced the statistical power to show a potential association between magnesium with markers of subclinical CVD. Because our study design was cross-sectional, the temporality and causality of the associations could not be concluded. Moreover, since our analysis was performed on a selected cross-sectional sample, generalizability to other populations might be limited.

## 5. Conclusions

Our results showed that serum and dietary magnesium were associated with some markers of subclinical CVD burden among participants without manifest cardiovascular disease, and thus may be implicated in cardiovascular disease development already at the subclinical stage. However, the findings were inconsistent and highlighted the importance to consider both dietary and serum magnesium since these entities do not correlate well. Larger, well-characterized, population-based studies are required to increase statistical power and confirm and extend our findings.

## Figures and Tables

**Table 1:** (**a**): Demographic and cardiovascular risk factors by serum magnesium status; (**b**): demographic and cardiovascular risk factors by dietary magnesium intake.

(**a**)				
	All	Mg ≤ 2.07 mg/dL	Mg > 2.07 mg/dL	*p* Value
	N = 396 (99.2%)	n = 181 (45.7%)	n = 215 (54.3%)	
Age (years)	56.34 (9.20)	57.04 (9.65)	55.75 (8.79)	0.167
Male sex	229 (57.8)	108 (59.7%)	121 (56.3%)	0.563
Weight (kg)	83.00 (16.63)	83.60 (15.96)	82.49 (17.19)	0.508
BMI (kg/m^2^)	28.10 (4.91)	28.21 (4.83)	28.00 (4.99)	0.67
Smoking				0.345
Never smoker	145 (36.6%)	67 (37.0%)	78 (36.3%)	
Ex-smoker	171 (43.2%)	83 (45.9%)	88 (40.9%)	
Smoker	80 (20.2%)	31 (17.1%)	49 (22.8%)	
Waist circumference (cm)	98.56 (14.37)	99.24 (14.17)	98.00 (14.55)	0.394
Systolic BP (mmHg)	120.54 (16.64)	122.60 (16.31)	118.81 (16.75)	0.024
Diastolic BP (mmHg)	75.24 (9.99)	75.93 (9.67)	74.67 (10.24)	0.514
Physically active	237 (59.8%)	112 (61.9%)	125 (58.1%)	0.523
Hypertension	133 (33.6%)	65 (35.9%)	68 (31.6%)	0.428
Glucose (mg/dl)	104.31 (22.63)	107.36 (25.04)	101.74 (20.08)	0.014
HbA1c (%)	5.57 (0.72)	5.63 (0.74)	5.52 (0.71)	0.121
Diabetes				0.022
No	242 (61.1%)	105 (58.0%)	137 (63.7%)	
Prediabetes	102 (25.8%)	43 (23.8%)	59 (27.4%)	
Diabetes	52 (13.1%)	33 (18.2%)	19 (8.8%)	
Total cholesterol (mg/dl)	218.05 (36.31)	215.12 (34.54)	220.51 (37.65)	0.141
HDL-C (mg/dl)	62.00 (17.68)	62.14 (17.62)	61.89 (17.76)	0.888
LDL-C (mg/dl)	139.68 (32.98)	136.71 (31.51)	142.17 (34.04)	0.101
Triglycerides (mg/dl)	131.41 (85.12)	133.42 (92.51)	129.73 (78.54)	0.668
eGFR (ml/min/1.73 m^2^)	86.62 (12.96)	86.18 (13.13)	87.00 (12.83)	0.531
Serum potassium (mmol/L)	4.29 (0.28)	4.30 (0.29)	4.27 (0.28)	0.298
Serum phosphate (mmol/L)	1.04 (0.15)	1.04 (0.15)	1.05 (0.15)	0.4
Diabetic medication	30 (7.6%)	23 (12.7%)	7 (3.3%)	0.001
Antihypertensive medication	100 (25.3%)	49 (27.1%)	51 (23.7%)	0.517
Lipid lowering medication	42 (10.6%)	21 (11.6%)	21 (9.8%)	0.669
Diuretics medication	54 (13.6%)	23 (12.7%)	31(14.4%)	0.728
Anticoagulant therapy	8 (2.0%)	4 (2.2%)	4 (1.9%)	1
(**b**)				
	**All**	**Dietary Mg ≤ 155.2 mg/1000 kcal/day**	**Dietary Mg > 155.2 mg/1000 kcal/day**	***p* Value**
	**N = 311 (77.9%)**	**n = 154 (49.5%)**	**n = 157 (50.5%)**	
Age (years)	56.39 (9.10)	55.86 (9.23)	56.91 (8.97)	0.311
Male sex	175 (56.3%)	118 (76.6%)	57 (36.3%)	<0.001
Weight (kg)	82.23 (16.60)	85.79 (15.81)	78.74 (16.67)	<0.001
BMI (kg/m^2^)	27.95 (4.97)	28.19 (4.64)	27.71 (5.27)	0.394
Smoking				0.495
Never smoker	115 (37.0%)	52 (33.8%)	63 (40.1%)	
Ex-smoker	136 (43.7%)	70 (45.5%)	66 (42.0%)	
Smoker	60 (19.3%)	32 (20.8%)	28 (17.8%)	
Waist circumference (cm)	97.99 (14.56)	100.86 (13.90)	95.18 (14.69)	0.001
Systolic BP (mmHg)	120.05 (16.36)	123.13 (16.31)	117.02 (15.89)	0.001
Diastolic BP (mmHg)	74.80 (9.90)	76.30 (9.97)	73.34 (9.64)	0.008
Physically active	189 (60.8%)	88 (57.1%)	101 (64.3%)	0.237
Hypertension	108 (34.7%)	60 (39.0%)	48 (30.6%)	0.151
Glucose (mg/dl)	103.44 (18.28)	105.61 (20.96)	101.31 (14.96)	0.038
HbA1c (%)	5.53 (0.59)	5.56 (0.63)	5.51 (0.54)	0.438
Diabetes				0.531
No	192 (61.7%)	93 (60.4%)	99 (63.1%)	
Prediabetes	83 (26.7%)	40 (26.0%)	43 (27.4%)	
Diabetes	36 (11.6%)	21 (13.6%)	15 (9.6%)	
Total cholesterol (mg/dl)	217.67 (36.18)	216.32 (37.37)	218.89 (35.05)	0.532
HDL-C (mg/dl)	62.63 (17.82)	58.89 (16.89)	66.29 (18.00)	<0.001
LDL-C (mg/dl)	139.31 (33.50)	139.25 (34.12)	139.36 (32.99)	0.977
Triglycerides (mg/dl)	127.65 (79.43)	144.12 (97.15)	111.49 (52.43)	<0.001
eGFR (ml/min/1.73 m^2^)	86.63 (13.10)	86.98 (13.34)	86.28 (12.88)	1
Energy intake (kcal/day)	1841.53 (414.39)	2004.73 (383.37)	1681.45 (380.79)	<0.001
Dietary calcium (mg/day)	763.29 (205.97)	739.29 (193.76)	786.83 (215.30)	<0.001
Dietary potassium (mg/day)	2532.28 (503.22)	2515.33 (470.74)	2548.91 (534.16)	0.557
Dietary phosphate (mg/day)	1111.75 (263.84)	1130.93 (244.04)	1092.94 (281.43)	0.205
Diabetic medication	23 (7.4%)	13 (8.4%)	10 (6.4%)	0.630
Antihypertensive medication	84 (27.0%)	45 (29.2%)	39 (24.8%)	0.458
Lipid lowering medication	34 (10.9%)	21 (13.6%)	13 (8.3%)	0.183
Diuretics medication	49 (15.8%)	22 (14.3%)	27 (17.2%)	0.583
Anticoagulant therapy	8 (2.6%)	4 (2.6%)	4 (2.5%)	1

Values are reported as mean (standard deviation) or n (%), unless otherwise indicated. BP, blood pressure; BMI, body mass index; eGFR, estimated glomerular filtration rate.

**Table 2:** (**a**): Imaging markers of subclinical cardiovascular disease by serum magnesium status. (**b**): Imaging markers of subclinical cardiovascular disease by dietary magnesium intake.

(a)				
	All	Mg ≤ 2.07 mg/dL	Mg > 2.07 mg/dL	*p* Value
Left Ventricular Function	N = 366 (91.7%)	n = 168 (45.9%)	n = 198 (54.1%)	
Early diastolic filling rate (mL/s)	226.11 (115.89)	223.95 (115.97)	227.93 (116.09)	0.744
Late diastolic filling rate (mL/s)	225.90 (109.28)	223.95 (113.18)	227.56 (106.11)	0.754
End diastolic volume (mL/m^2^)	66.14 (14.90)	66.50 (16.21)	65.84 (13.73)	0.673
End systolic volume (mL/m^2^)	20.73 (8.65)	20.87 (9.67)	20.60 (7.70)	0.764
Stroke volume (mL/m^2^)	45.43 (9.42)	45.65 (10.06)	45.24 (8.85)	0.682
Cardiac output (mL/min/m^2^)	3009.05 (586.74)	3050.06 (617.36)	2974.25 (558.66)	0.219
Ejection fraction (%)	69.37 (7.78)	69.52 (8.45)	69.25 (7.18)	0.741
Peak ejection rate (mL/s)	354.70 (132.64)	352.74 (136.76)	356.36 (129.37)	0.795
Myocardial mass (g/m^2^)	71.46 (13.37)	72.46 (13.80)	70.61 (12.97)	0.188
LGE	11 (3.0%)	3 (1.8%)	8 (4.0%)	0.341
Remodeling index (g/mL/m^2^)	0.58 (0.15)	0.59 (0.17)	0.57 (0.14)	0.418
Mean diastolic thickness (mm/m^2^)	4.85 (0.67)	4.88 (0.72)	4.82 (0.61)	0.33
**Right ventricular function**	**N = 336 (84.2%)**	**n = 85 (25.3%)**	**n = 251 (74.7%)**	
End diastolic volume (mL/m^2^)	84.72 (17.46)	87.63 (18.54)	83.73 (17.00)	0.075
End systolic volume (mL/m^2^)	40.33 (11.78)	42.70 (12.16)	39.52 (11.56)	0.031
Stroke volume (mL/m^2^)	44.43 (9.11)	44.95 (9.45)	44.25 (9.00)	0.539
Cardiac output (mL/min/m^2^)	2938.74 (574.20)	3004.43 (575.29)	2916.49 (573.27)	0.223
Ejection fraction (%)	52.85 (7.01)	51.80 (6.74)	53.21 (7.08)	0.109
**Carotid plaque**	**N = 248 (62.2%)**	**n = 76 (30.6%)**	**n = 172 (69.4%)**	
Presence of plaque	50 (20.2%)	12 (15.8%)	38 (22.1)	0.333
Presence of plaque type				0.416
AHA type I	198 (79.8%)	64 (84.2%)	134 (77.9)	
AHA type III	34 (13.7%)	7 (9.2%)	27 (15.7)	
AHA type V	10 (4.0%)	4 (5.3%)	6 (3.5)	
AHA type VI or VII	6 (2.4%)	1 (1.3%)	5 (2.9)	
Wall thickness left (mm)	0.75 (0.11)	0.76 (0.13)	0.74 (0.10)	0.112
Wall thickness right (mm)	0.76 (0.10)	0.78 (0.10)	0.75 (0.10)	0.018
(**b**)				
	**All**	**Dietary Mg ≤ 155.2 mg/1000 kcal/day**	**Dietary Mg > 155.2 mg/1000 kcal/day**	***p* Value**
**Left Ventricular Function**	**N = 287 (91.8%)**	**n = 141 (49.1%)**	**n = 146 (50.9%)**	
Early diastolic filling rate (mL/s)	229.48 (115.39)	226.99 (110.31)	231.87 (120.42)	0.721
Late diastolic filling rate (mL/s)	227.74 (110.88)	226.97 (118.89)	228.48 (102.96)	0.908
End diastolic volume (mL/m^2^)	66.65 (14.81)	65.83 (14.37)	67.45 (15.23)	0.355
End systolic volume (mL/m^2^)	20.73 (8.08)	20.48 (7.81)	20.97 (8.35)	0.607
Stroke volume (mL/m^2^)	45.94 (9.43)	45.38 (9.17)	46.47 (9.67)	0.327
Cardiac output (mL/min/m^2^)	3041.45 (574.70)	3047.67 (561.30)	3035.44 (589.22)	0.857
Ejection fraction (%)	69.53 (7.27)	69.46 (6.93)	69.60 (7.61)	0.875
Peak ejection rate (mL/s)	356.40 (133.62)	361.26 (136.96)	351.71 (130.61)	0.546
Myocardial mass (g/m^2^)	70.71 (12.72)	73.57 (12.56)	67.94 (12.29)	<0.001
LGE	9 (3.1%)	3 (2.1%)	6 (4.1%)	0.532
Remodeling index (g/mL/m^2^)	0.57 (0.14)	0.58 (0.15)	0.56 (0.13)	0.091
Mean diastolic thickness (mm/m^2^)	4.81 (0.63)	4.88 (0.65)	4.75 (0.61)	0.090
**Right ventricular function**	**N = 263 (84.0%)**	**n = 128 (48.7%)**	**n = 135 (51.3%)**	
End diastolic volume (mL/m^2^)	85.66 (17.65)	85.92 (17.27)	85.41 (18.07)	0.817
End systolic volume (mL/m^2^)	40.52 (12.03)	41.36 (11.94)	39.72 (12.10)	0.271
Stroke volume (mL/m^2^)	45.18 (9.00)	44.58 (8.65)	45.74 (9.32)	0.296
Cardiac output (mL/min/m^2^)	2974.97 (574.37)	3000.75 (561.10)	2950.51 (587.71)	0.118
Ejection fraction (%)	53.21 (6.89)	52.37 (6.83)	54.01 (6.88)	<0.001
**Carotid plaque**	**N = 188 (60.1%)**	**n = 100 (53.2%)**	**n = 88 (46.8%)**	
Presence of plaque	41 (21.8%)	23 (23.0%)	18 (20.5%)	0.807
Presence of plaque type				0.265
AHA type I	147 (78.2%)	77 (77.0%)	70 (79.5%)	
AHA type III	28 (14.9%)	15 (15.0%)	13 (14.8%)	
AHA type V	7 (3.7%)	6 (6.0%)	1 (1.1%)	
AHA type VI or VII	6 (3.2%)	2 (2.0%)	4 (4.5%)	
Wall thickness left (mm)	0.75 (0.11)	0.75 (0.12)	0.74 (0.10)	0.463
Wall thickness right (mm)	0.76 (0.10)	0.76 (0.10)	0.75 (0.11)	0.248

Values are reported as the mean (SD), n (%), unless otherwise indicated. LGE: Late Gadolinium Enhancement, AHA: American Heart Association

**Table 3:** Results of multivariable-adjusted models for the relation between serum and dietary magnesium and imaging markers of subclinical cardiovascular disease.

	Serum Magnesium			Dietary Magnesium		
	Estimate	95% CI	*p* Value	Estimate	95% CI	*p* Value
Left Ventricular Function		N = 366			N = 287	
Early diastolic filling rate (mL/s)	−3.29	(−14.24, 7.67)	0.556	0.17	(−0.217, 0.556)	0.388
Late diastolic filling rate (mL/s)	−0.18	(−11.17, 10.81)	0.975	0.05	(−0.347, 0.453)	0.794
End diastolic volume (mL/m^2^)	−0.86	(−2.32, 0.58)	0.239	0.06	(0.014, 0.114)	0.013
End systolic volume (mL/m^2^)	−0.07	(−0.94, 0.79)	0.871	0.04	(0.008, 0.065)	0.011
Stroke volume (mL/m^2^)	−0.81	(−1.72, 0.10)	0.082	0.03	(−0.006, 0.059)	0.104
Cardiac output (mL/min/m^2^)	−52.73	(−111.58, 6.11)	0.078	1.42	(−0.631, 3.471)	0.174
Ejection fraction (%)	−0.38	(−1.16, 0.41)	0.341	−0.02	(−0.048, 0.004)	0.101
Peak ejection rate (mL/s)	5.38	(−7.66, 18.42)	0.418	−0.22	(−0.685, 0.253)	0.365
Myocardial mass (g/m^2^)	0.003	(−1.17, 1.18)	0.995	−0.01	(−0.052, 0.028)	0.546
LGE	OR 3.06	(1.27, 8.32)	0.018	OR 1.01	(0.988, 1.036)	0.434
Remodeling index (g/mL/m^2^)	0.005	(−0.009, 0.019)	0.479	−0.001	(−0.001. −0.0002)	0.004
Mean diastolic thickness (mm/m^2^)	0.02	(−0.04, 0.08)	0.565	−0.002	(−0.004, −0.0002)	0.029
**Right ventricular function**		**(N = 336)**			**(N = 236)**	
End diastolic volume (mL/m^2^)	−1.74	(−3.50, 0.02)	0.053	0.05	(−0.011, 0.114)	0.105
End systolic volume (mL/m^2^)	−1.21	(−2.39, −0.04)	0.043	0.03	(−0.011, 0.071)	0.171
Stroke volume (mL/m^2^)	−0.53	(−1.47, 0.41)	0.268	0.02	(−0.011, 0.056)	0.187
Cardiac output (mL/min/m^2^)	−32.62	(−93.93, 28.70)	0.296	1.51	(−0.852, 3.474)	0.169
Ejection fraction (%)	0.33	(−0.38, 1.04)	0.362	−0.01	(−0.034, 0.014)	0.441
**Carotid plaque**		**(N = 248)**			**(N = 188)**	
Presence of plaque	OR 1.62	(1.07, 2.56)	0.033	OR 0.99	(0.975,1.000)	0.056
Presence of plaque type	OR 1.58	(1.19, 2.11)	0.002	OR 0.99	(0.980, 0.996)	0.004
Wall thickness left (mm)	−0.003	(−0.016, 0.009)	0.66	−0.0001	(−0.0006, 0.0003)	0.562
Wall thickness right (mm)	−0.008	(−0.019, 0.004)	0.211	−0.0001	(−0.0006, 0.0002)	0.381

All models are adjusted for age, sex, BMI, systolic blood pressure, and diabetes. Daily caloric intake was additionally adjusted for models with dietary magnesium. Serum cholesterol and smoking status were additionally adjusted for the association between serum magnesium and LGE, presence and type of plaque. Estimates represent β-estimates from linear regression or OR from logistic regression.

## Data Availability

The informed consent given by KORA study participants does not cover data posting in public databases. However, data are available upon request from the KORA database by means of a project agreement. Requests should be sent to kora.passt@helmholtz-muenchen.de and are subject to approval by the KORA Board.

## Supplementary Materials

The following supporting information can be downloaded at: https://www.mdpi.com/article/10.3390/nu14234954/s1, Table S1: Demographic and cardiovascular characteristics of excluded and included participants for the respective analyses. Table S2: Demographic and cardiovascular risk factors by serum and dietary magnesium. Table S3: Correlation between serum and dietary magnesium with cardiovascular risk factors and imaging markers of subclinical cardiovascular disease, assessed by Spearman correlation. Table S4: Imaging markers of subclinical cardiovascular disease by serum and dietary magnesium.
