# Supplementary material for: Subclinical Cardiovascular Disease Markers in Relation to Serum and Dietary Magnesium in Individuals from the General Population: The KORA-MRI Study
Source: Nutrients. 2022 Nov 22;14(23):4954. doi: 10.3390/nu14234954 (PMC9741061; doi:10.3390/nu14234954)
Supplement: Supplementary file 1 [file nutrients-14-04954-s001.zip › nutrients-2027131-supplementary.pdf]

Table S1: Demographic and cardiovascular characteristics of excluded and included participants for the respective analyses.

	Serum magnesium			Dietary magnesium		
	excluded	included	P value	excluded	included	P value
**LVF**	**(N = 36)**	**(N = 363)**		**(N = 114)**	**(N = 285)**	
BMI (kg/m2)	30.00 (5.92)	27.95 (4.78)	0.017	28.98 (5.11)	27.79 (4.80)	0.029
Waist circumference (cm)	103.54(16.62)	98.12(14.01)	0.030	101.13(14.63)	97.60 (14.11)	0.026
Hypertension	19 (52.8%)	117 (32.2%)	0.022	………..	………..	………
Diabetes	12 (33.3%)	42 (11.6%)	0.001	26 (22.8%)	28 (9.8%)	0.002
Hypertensive medication	17 (47.2%)	85 (23.4%)	0.003	……….	…………	………
Diabetic medication	7 (19.4%)	25 (6.9%)	0.020	………..	………...	………
HbA1c (%)	………..	…………	………	5.73 (1.03)	5.52 (0.57)	0.008
**RVF**	**(N = 66)**	**(N = 333)**		**(N = 138)**	**(N = 261)**	
Weight (Kg)	…………	…………	………	85.61 (16.52)	81.63 (16.45)	0.022
BMI (kg/m2)	…………	…………	………	28.81 (4.89)	27.77 (4.90)	0.046
Waist circumference (cm)	…………	…………	………	101.06(14.00)	97.31 (14.36)	0.013
Systolic BP (mmHg)	…………	…………	………	123.59(16.85)	119.12(16.49)	0.011
Triglycerides (mg/dl)	…………	…………	………	145.95(95.44)	124.09(77.83)	0.014
Diuretics med	…………	…………	………	11 (8.0%)	43 (16.5%)	0.027
**Carotid plaque**	**(N = 154)**	**(N = 245)**		**(N = 213)**	**(N = 186)**	
Male	77 (50.0%)	153 (62.4)	0.019	…………	…………	………
BMI (kg/m2)	29.06 (5.63)	27.55 (4.32)	0.003	28.70 (5.22)	27.48 (4.47)	0.013
Waist circumference (cm)	…………	…………	………	99.98 (14.96)	97.05 (13.45)	0.041
Diabetes	14 (9.1%)	40 (16.3%)	<0.001	28 (13.1%)	26 (14.0%)	0.010
Diabetic medication	6 (3.9%)	26 (10.6)	0.027	…………	…………	………
Serum potassium (mmol/l)	4.24 (0.26)	4.32 (0.29)	0.005	…………	…………	………

Only variables with significant differences ($p<0.05$) between included and excluded participants are presented. Values are reported as the mean (SD) or n (%), unless otherwise specified.
BP, blood pressure; BMI, body mass index

Table S2: Demographic and cardiovascular risk factors by serum and dietary magnesium

	All	Low serum & dietary Mg	Low serum & high dietary Mg	High serum & low dietary Mg	High serum & dietary Mg	P value
	**N = 311 (77.9%)**	**N = 75 (24.1%)**	**N = 66 (21.2%)**	**N = 79 (25.4%)**	**N = 91 (29.3%)**	
Age (years)	56.39 (9.10)	57.08 (9.07)	57.52 (9.86)	54.71 (9.30)	56.47 (8.30)	0.247
Male sex	175 (56.3%)	56 (74.7%)	27 (40.9%)	62 (78.5%)	30 (33.0%)	**<0.001**
Weight (kg)	82.23 (16.60)	85.65 (17.00)	80.31 (16.35)	85.93 (14.71)	77.60 (16.89)	**0.002**
BMI (kg/m2)	27.95 (4.97)	28.25 (5.04)	28.16 (5.31)	28.14 (4.27)	27.39 (5.25)	0.650
Smoking						0.584
Never smoker	115 (37.0%)	24 (32.0%)	31 (47.0%)	28 (35.4%)	32 (35.2%)	
Ex-smoker	136 (43.7%)	37 (49.3%)	25 (37.9%)	33 (41.8%)	41 (45.1%)	
Smoker	60 (19.3%)	14 (18.7%)	10 (15.2%)	18 (22.8%)	18 (19.8%)	
Waist circumference (cm)	97.99 (14.56)	101.29 (15.19)	96.51 (14.08)	100.44 (12.64)	94.22 (15.12)	**0.005**
Systolic BP (mmHg)	120.05 (16.36)	124.89 (16.62)	119.49 (14.39)	121.46 (15.94)	115.23 (16.75)	**0.002**
Diastolic BP (mmHg)	74.80 (9.90)	77.08 (10.59)	73.97 (8.86)	75.55 (9.35)	72.88 (10.20)	**0.039**
Physically active	189 (60.8%)	44 (58.7%)	39 (59.1%)	44 (55.7%)	62 (68.1%)	0.371
Hypertension	108 (34.7%)	33 (44.0%)	21 (31.8%)	27 (34.2%)	27 (29.7%)	0.615
Glucose (mg/dl)	103.44 (18.28)	110.73 (25.44)	102.73 (15.70)	100.75 (14.11)	100.29 (14.39)	**0.001**
HbA1c (%)	5.53 (0.59)	5.70 (0.78)	5.53 (0.61)	5.43 (0.41)	5.50 (0.49)	**0.034**
Diabetes						**0.037**
No	192 (61.7%)	42 (56.0%)	40 (60.6%)	51 (64.6%)	59 (64.8%)	
Prediabetes	83 (26.7%)	17 (22.7%)	16 (24.2%)	23 (29.1%)	27 (29.7%)	
Diabetes	36 (11.6%)	16 (21.3%)	10 (15.2%)	5 (6.3%)	5 (5.5%)	
Total cholesterol (mg/dl)	217.67 (36.18)	158.52 (117.74)	212.85 (32.56)	216.30 (38.57)	223.27 (36.29)	0.313
HDL-C (mg/dl)	62.63 (17.82)	58.28 (17.40)	66.07 (16.46)	59.47 (16.48)	66.45 (19.13)	**0.003**
LDL-C (mg/dl)	139.31 (33.50)	137.96 (33.09)	108.92 (52.99)	140.48 (35.23)	143.05 (33.79)	0.417
Triglycerides (mg/dl)	127.65 (79.43)	142.52 (107.30)	132.14 (91.27)	130.44 (70.52)	113.35 (52.24)	**<0.001**
eGFR (ml/min/1.73 m2)	86.63 (13.10)	87.86 (14.04)	85.08 (13.01)	86.15 (12.68)	87.15 (12.79)	0.608
Energy intake (kcal/day)	1841.53 (414.39)	1949.80 (354.41)	1698.68 (374.16)	2056.87 (404.33)	1668.96 (387.11)	**<0.001**
Dietary calcium (mg/day)	763.29 (205.97)	724.21 (185.42)	803.69 (247.47)	753.60 (201.50)	774.60 (189.09)	0.127
Dietary potassium (mg/day)	2532.28 (503.22)	2462.53 (447.17)	2587.66 (560.32)	2565.46 (489.62)	2520.81 (515.67)	0.454
Dietary phosphate (mg/day)	1111.75 (263.84)	1094.27 (229.00)	1111.44 (297.10)	1165.73 (254.06)	1079.52 (270.37)	0.172
Diabetic medication	23 (7.4%)	11 (14.7%)	8 (12.1%)	2 (2.5%)	2 (2.2%)	**0.003**
Antihypertensive medication	84 (27.0%)	24 (32.0%)	18 (27.3%)	21 (26.6%)	21 (23.1%)	0.643
Lipid lowering medication	34 (10.9%)	14 (18.7%)	4 (6.1%)	7 (8.9%)	9 (9.9%)	0.083
Diuretics medication	49 (15.8%)	9 (12.0%)	10 (15.2%)	13 (16.5%)	17 (18.7%)	0.698
Anticoagulant medication	8 (2.6%)	3 (4.0%)	1 (1.5%)	1 (1.3%)	3 (3.3%)	0.652

Values are reported as the mean (SD) or n (%), unless otherwise specified. BP, blood pressure; BMI, body mass index; eGFR, estimated glomerular filtration rate

Table S3: Correlation between serum and dietary magnesium with cardiovascular risk factors and imaging markers of subclinical cardiovascular disease, assessed by Spearman correlation.

	**Serum magnesium (mg/dl)**		**Dietary magnesium (mg/day)**	
	**r coefficient**	**p value**	**r coefficient**	**p value**
**Covariates**	**(N= 394)**		**(N = 311)**	
Age (years)	-0.082	0.103	-0.104	0.068
Weight (kg)	-0.024	0.635	0.193	**<0.001**
BMI (kg/m2)	-0.007	0.893	-0.074	0.192
Waist circumference (cm)	-0.030	0.547	0.069	0.225
Systolic BP (mmHg)	-0.149	**0.003**	0.125	**0.028**
Diastolic BP (mmHg)	-0.094	0.061	0.095	0.094
Glucose (mg/dl)	-0.147	**0.003**	0.003	0.953
HbA1c (%)	-0.053	0.293	-0.135	**0.017**
Total cholesterol (mg/dl)	0.103	**0.04**	-0.053	0.354
HDL-C (mg/dl)	-0.037	0.465	-0.033	0.561
LDL-C (mg/dl)	0.131	**0.009**	-0.022	0.701
Triglycerides (mg/dl)	0.003	0.952	0.013	0.810
eGFR (ml/min/1.73 m2)	0.013	0.790	0.02	0.730
Serum potassium (mmol/l)	-0.027	0.596	....	....
Serum phosphate (mmol/l)	0.101	**0.045**	....	....
Serum magnesium (mmol/l)	1	0.000	0.038	0.501
Energy intake (kcal/day)	....	....	0.845	**<0.001**
Dietary calcium (mg/day)	....	....	0.580	**<0.001**
Dietary potassium (mg/day)	....	....	0.899	**<0.001**
Dietary phosphate (mg/day)	....	....	0.914	**<0.001**
**Left ventricular function**	**(N = 366)**		**(N = 287)**	
Early diastolic filling rate (ml/s)	0.028	0.598	0.113	0.055
Late diastolic filling rate (ml/s)	0.017	0.746	0.088	0.136
End diastolic volume (ml/m2)	-0.030	0.564	0.192	**0.001**
End systolic volume (ml/m2)	-0.009	0.860	0.205	**<0.001**
Stroke volume (ml/m2)	-0.015	0.775	0.133	0.024
Cardiac output (ml/min/m2)	-0.068	0.195	0.172	0.003
Ejection fraction (%)	-0.018	0.727	-0.137	**0.021**
Peak ejection rate (ml/s)	-0.022	0.696	-0.166	**0.005**
Myocardial mass (g/m2)	-0.058	0.267	0.224	**<0.001**
Remodeling index (g/ml/m2)	0.008	0.877	-0.185	**0.002**
Mean diastolic thickness (mm/m2)	-0.068	0.195	-0.120	**0.042**
**Right ventricular function**	**(N = 334)**		**(N = 263)**	
End diastolic volume (ml/m2)	-0.089	0.103	0.256	**<0.001**
End systolic volume (ml/m2)	-0.134	0.014	0.327	**<0.001**
Stroke volume (ml/m2)	-0.008	0.878	0.098	0.112
Cardiac output (ml/min/m2)	-0.072	0.187	0.168	**0.006**
Ejection fraction (%)	0.122	0.025	-0.285	**<0.001**
**Carotid plaque**	**(N = 248)**		**(N = 188)**	
Wall thickness left (mm)	-0.044	0.488	0.001	0.994
Wall thickness right (mm)	-0.132	**0.037**	-0.046	0.530

Table S4: Imaging markers of subclinical cardiovascular disease by serum and dietary magnesium

	All	Low serum & dietary Mg	Low serum & high dietary Mg	High serum & low dietary Mg	High serum & dietary Mg	P value
**Left ventricular function**	**N = 287 (91.7%)**	**N = 71 (24.7%)**	**N = 61 (21.3%)**	**N = 70 (24.4%)**	**N = 85 (29.6%)**	
Early diastolic filling rate (ml/s)	229.48 (115.39)	233.10 (117.45)	217.20 (107.66)	220.80 (103.04)	242.40 (128.39)	0.529
Late diastolic filling rate (ml/s)	227.74 (110.88)	229.09 (125.44)	219.41 (104.52)	224.82 (112.71)	234.99 (101.95)	0.859
End diastolic volume (ml/m2)	66.65 (14.81)	66.42 (16.49)	66.42 (16.49)	64.36 (13.00)	68.18 (14.31)	0.437
End systolic volume (ml/m2)	20.73 (8.08)	20.95 (8.62)	20.76 (8.55)	20.00 (6.93)	21.12 (8.26)	0.845
Stroke volume (ml/m2)	45.94 (9.43)	46.36 (9.74)	45.66 (10.62)	44.39 (8.51)	47.05 (8.94)	0.352
Cardiac output (ml/min/m2)	3041.45 (574.70)	3116.56 (609.98)	3046.41 (657.54)	2977.80 (501.93)	3027.58 (538.81)	0.549
Ejection fraction (%)	69.53 (7.27)	69.61 (7.38)	69.46 (7.97)	69.31 (6.50)	69.69 (7.38)	0.990
Peak ejection rate (ml/s)	356.40 (133.62)	368.23 (151.20)	331.74 (117.99)	354.20 (121.53)	366.04 (137.87)	0.379
Myocardial mass (g/m2)	70.71 (12.72)	74.96 (12.40)	67.75 (12.51)	72.16 (12.67)	68.08 (12.20)	**0.001**
LGE	9 (3.1%)	2 (2.8%)	1 (1.6%)	1 (1.4%)	5 (5.9%)	0.354
Remodeling index (g/ml/m2)	0.57 (0.14)	0.59 (0.15)	0.56 (0.16)	0.58 (0.15)	0.55 (0.11)	0.363
Mean diastolic thickness (mm/m2)	4.81 (0.63)	4.94 (0.69)	4.74 (0.64)	4.81 (0.60)	4.76 (0.58)	0.216
**Right ventricular function**	**N = 263 (84%)**	**N = 65 (24.7%)**	**N = 52 (19.8%)**	**N = 63 (23.9%)**	**N = 83 (31.6%)**	
End diastolic volume (ml/m2)	85.66 (17.65)	88.17 (18.09)	86.85 (19.94)	83.59 (16.21)	84.51 (16.85)	0.431
End systolic volume (ml/m2)	40.52 (12.03)	43.07 (12.45)	41.12 (12.15)	39.59 (11.21)	38.84 (12.06)	0.169
Stroke volume (ml/m2)	45.18 (9.00)	45.16 (8.82)	45.74 (10.55)	43.99 (8.49)	45.74 (8.52)	0.653
Cardiac output (ml/min/m2)	1561.98(1314.1)	3043.38 (608.65)	3022.10 (675.59)	2956.78 (508.55)	2905.67 (524.66)	0.470
Ejection fraction (%)	53.21 (6.89)	51.71 (6.71)	53.08 (6.41)	53.05 (6.94)	54.59 (7.14)	0.090
**Carotid plaque**	**N = 188 (60.1%)**	**N = 57 (30.3%)**	**N= 40 (21.3%)**	**N = 43 (22.9)**	**N = 48 (25.5%)**	
Presence of plaque	41 (21.8%)	11 (19.3%)	6 (15.0%)	12 (27.9%)	12 (25.0%)	0.471
Presence of plaque type						0.135
AHA type I	147 (78.2%)	46 (80.7%)	34 (85.0%)	31 (72.1%)	36 (75.0%)	
AHA type III	28 (14.9%)	7 (12.3%)	5 (12.5%)	8 (18.6%)	8 (16.7%)	
AHA type V	7 (3.7%)	2 (3.5%)	1 (2.5%)	4 (9.3%)	0 (0.0%)	
AHA type VI or VII	6 (3.2%)	2 (3.5%)	0 (0.0%)	0 (0.0%)	4 (8.3%)	
Wall thickness left (mm)	0.75 (0.11)	0.77 (0.13)	0.75 (0.09)	0.74 (0.09)	0.74 (0.11)	0.444
Wall thickness right (mm)	0.76 (0.10)	0.79 (0.11)	0.76 (0.10)	0.73 (0.08)	0.73 (0.11)	**0.016**

Values are reported as the mean (SD), n (%), unless otherwise indicated
